# The Tale of a Rubbery White Clot: Poorly Differentiated Spindle Cell Sarcoma Presenting As Superior Vena Cava Syndrome

**DOI:** 10.7759/cureus.25071

**Published:** 2022-05-17

**Authors:** Swe Swe Hlaing, Emeka Ugwuegbulem, Kyle Haddock, Ei Ei Thwe, Yacoub Faroun

**Affiliations:** 1 Department of Internal Medicine, Crozer-Chester Medical Center, Upland, USA; 2 Department of Internal Medicine, St Luke's Hospital, Bethlehem, USA; 3 Department of Oncology, St Luke's Hospital, Bethlehem, USA

**Keywords:** spindle cell mesenchymal tumor, hemato-oncology, oncologic emergencies, spindle cell sarcoma, superior vena cava (svc) syndrome

## Abstract

Sarcoma is an uncommon neoplasm of mesenchymal origin (1). The presentation is usually vague. It may present as a mass in the thigh or retroperitoneum, with resultant pain or paresthesia of the affected area. The diagnosis is very challenging due to its indistinct presentation. The prognosis remains poor due to delays in diagnosis and few available therapeutic options. We herein report the first case of superior vena cava (SVC) syndrome caused by spindle cell sarcoma.

## Introduction

Sarcoma is a heterogeneous collection of malignant tumors of mesenchymal origin that is extremely rare. It accounts for less than 1% of all adult cancers [1]. The cause of most sarcomas is unknown. Genetic predispositions [2], such as Li-Fraumani syndrome, neurofibromatosis type 1, and retinoblastoma; radiation therapy or chemotherapy exposure; carcinogen exposure; chronic irritation, lymphedema, human immunodeficiency virus, and human herpesvirus 8, are some of the known correlations. A progressive expansion of a painless lump, mainly in the thigh and retroperitoneum, is the most prevalent presenting symptom of sarcoma. Only a few individuals have complained of discomfort, paresthesia, and edema in their extremities as a result of tumor compression, as well as constitutional symptoms like fever, night sweats, and weight loss. The thigh, buttock, groin, upper extremities, chest, retroperitoneum, and head and neck are the most typically affected sites of sarcoma [3]. To the best of our knowledge, superior vena cava (SVC) syndrome from spindle cell sarcoma has never been reported.

## Case presentation

A 63-year-old male with a past medical history of heroin and tobacco abuse, coronary artery disease, hepatitis C, hypertension, and hyperlipidemia presented with worsening facial and upper extremity swelling for two days. He also had dysphagia to liquids without obvious drooling or stridor. His medication history was noted for the recent initiation of angiotensin-converting enzyme inhibitors (ACEI). On the exam, his heart rate was 60/min, oxygen saturation was 96% on room air, respiratory rate was 14/min and blood pressure was 80/60 mmHg. The blood pressure improved to 100/60 mmHg after the fluid boluses. Later he developed worsening hoarseness of voice and became apneic during sleep. He was intubated eventually for airway protection. His initial white blood cell level was 18.96/µL and hemoglobin level was 10 g/dL and serum albumin level was 3 g/dL. His initial working diagnosis was ACEI-induced angioedema and thus he received one unit of fresh frozen plasma.

**Figure 1:**
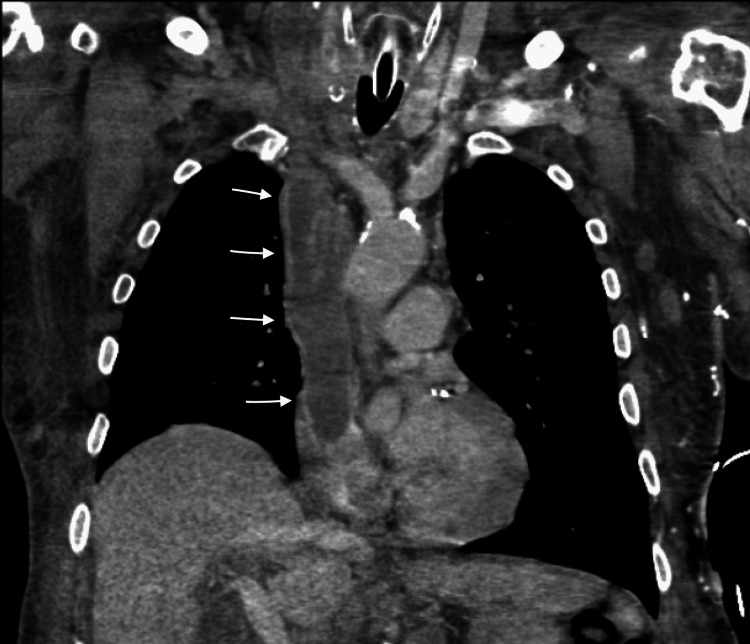
Coronal chest CT scan with arrows showing thrombus in SVC extending to right atrium

**Figure 2:**
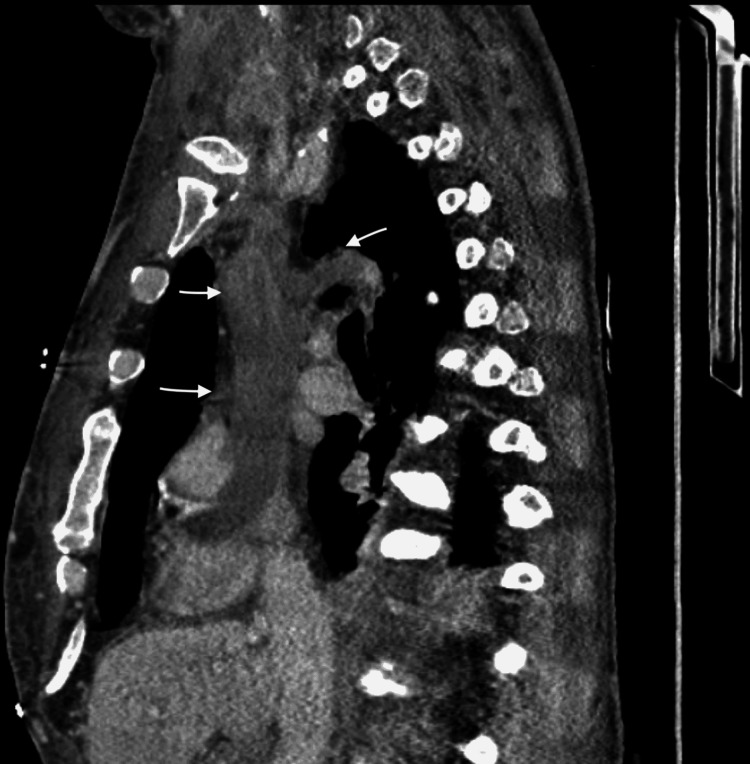
Sagittal chest CT scan with arrows showing thrombus in SVC and azygos vein

However, the CT chest showed a very large thrombus in the right atrium, SVC, bilateral subclavian veins, and azygos veins (Figures 1, 2). The patient was started on anticoagulation with intravenous heparin believing it is most likely the thrombus causing him to present with SVC syndrome. Given the extensive nature of the thrombus, the plan was to proceed with interventional radiology (IR) directed thrombectomy. During the large bore thrombectomy, it revealed hard white tissue-like structure with internal vascularity. There is extensive hard mass throughout the SVC with occlusion of the SVC and bilateral subclavian veins. Limited transthoracic echocardiography during the procedure revealed mass infiltrating the right atrium with at least a 2.4 cm component reverberating between the right atrium and right ventricle across the valve.

Intraprocedural frozen sections of the hard white material showed a preliminary report compatible with “spindle cell neoplasm.” The final pathology report of the tumor showed undifferentiated spindle cell sarcoma. The sample showed focally irregular tumor cell nuclei, scattered mitotic figures, negative immunostains for MDM2, CDK4, pan keratin, desmin, and S100 protein. But there was multifocal nuclear positivity for HMGA2 located on chromosome 12q.

He was not a surgical candidate since the tumor thrombus had spread into the left subclavian vein and was close to the azygos vein. Given his very poor condition requiring ventilator support, palliative chemotherapy and radiotherapy were ruled ineffective. Following a meeting with the patient's family, the family decided to opt for comfort care. And the patient was extubated with no further intensive treatment.

## Discussion

The primary spindle cell sarcoma is an extremely rare tumor with a poor prognosis and generally presents as a painless lump. SVC syndrome caused by undifferentiated spindle cell sarcoma has never been reported in the literature worldwide. In this case, we described undifferentiated spindle cell sarcoma with a very high degree of malignancy causing SVC syndrome. 

First, undifferentiated spindle cell sarcomas are difficult to diagnose and there are no invasive criteria for the diagnosis [4]. CT scan is the primary imaging modality but its effectiveness to differentiate benign and malignant tumors remains a challenge. 

Second, unlike other malignancies, spindle cell sarcoma can manifest in a variety of ways. Our patient presented to the hospital with nonspecific swelling of the face and upper extremities. Angioedema after recent ACEI initiation was the first working diagnosis. Later, a CT scan of the chest showed a large potential thrombus in SVC. It was extending from the right atrium, SVC to the left subclavian and azygos veins. This gave the idea that the thrombus had caused SVC syndrome. As the attempted IR-guided thrombectomy failed, the biopsy of the mass was obtained and it confirmed the patient's diagnosis of spindle cell sarcoma [5].

Finally, atypical SVC syndrome caused by a tumor thrombus in the SVC combined with a fast progressive clinical complaint should alert the physicians to the possibility of a more aggressive or malignant condition. Surgical excision has been suggested as the best therapeutic option. Endovascular stent placement was not recommended due to the aggressive nature of the tumor growth. Radiation and chemotherapy have limited effects [6]. Unfortunately, in our case, the patient lost his life-prolonging therapy.

## Conclusions

This case is very unique because the discovery of SVC syndrome was in a patient with spindle cell sarcoma. It was most commonly misdiagnosed due to its unfamiliarity. There is no standard treatment available for spindle cell sarcoma. Clinicians should have a broad differential when patients are presented with SVC syndrome. It highlights and adds to a growing body of literature regarding spindle cell sarcoma.
